# Effectiveness of probiotics on COVID-19 prevention and treatment against mild COVID-19 in outpatient care: A systematic review

**DOI:** 10.1177/02601060251378200

**Published:** 2025-09-26

**Authors:** Chung Hang Hannah Chau, Denes Stefler, Michelle Man Sum Szeto

**Affiliations:** 1 Institute of Epidemiology and Health Care, 384711 University College London, London, UK; 2 Nuffield Department of Population Health, 227951 University of Oxford, Oxford, UK

**Keywords:** COVID-19, probiotics, prophylaxis, microbiome, lactobacilli, bifidobacteria

## Abstract

**Background:**

In previous research, probiotics have shown to be beneficial in preventing and limiting the progress of upper respiratory infections. Their effectiveness in relation to coronavirus disease 2019 (COVID-19) has been investigated mainly in hospitalized patients, and less so among outpatients who constitute majority of COVID-19 cases.

**Aim:**

This systematic review evaluates the available evidence regarding the effectiveness of probiotic use on prevention and treatment of COVID-19 among patients with mild symptoms in outpatient settings.

**Methods:**

PubMed, Embase and Cochrane Library were searched for studies from their inception to May 2024, restricting to randomized controlled trials and before-and-after studies. The primary outcomes were infection incidence and complete remission rate. Cochrane risk-of-bias tool (RoB 2.0) and risk of bias in non-randomized studies of interventions tool (ROBINS-I) were used to assess the risk of bias. The Grading of Recommendations, Assessment, Development, and Evaluations approach was performed to assess the certainty of the evidence.

**Results:**

Eight randomized controlled trials and one pre-post study on 1235 participants were included. Four studies had low risk of bias. Probiotics were effective in reducing the incidence of COVID-19 upon exposure and accelerating the symptomatic remission of mild COVID-19 with less systemic symptoms. Overall, the certainty of evidence on both primary outcomes was moderate. Comorbidities and old ages were found to be significant confounders. Probiotics demonstrated significant immunomodulatory and humoral effects in the nasopharyngeal cavity.

**Conclusion:**

These results suggest that probiotics are effective at preventing COVID-19 and support faster recovery from mild COVID-19 among individuals seeking for outpatient care. People with comorbidities, that is, metabolic disorder and elderly benefit the most from probiotics supplements.

## Background

Coronavirus disease 2019 (COVID-19) remains to be a major public health challenge worldwide. As of 14 July 2024, there are 776 million confirmed cases of COVID-19 with over 7 million deaths (World Health Organization, 2024b, 2024c). Although vaccines are promising preventive treatment, their efficacy could be weakened by ribonucleic acid mutations of coronavirus, similar to that of influenza (Nguyen et al., 2022). In fact, over 90% of COVID-19 cases are outpatients with mild-to-moderate symptoms (Dugas et al., 2021) and early antiviral treatment is the existing key to reduce disease progression. However, current marketed antiviral drugs and dexamethasone steroid therapy are difficult to be administered in outpatient settings and of limited clinical values in mild disease (Li et al., 2023; Xu et al., 2020). Therefore, the future trend of public health lies on primary prevention, aiming to prevent development and spread of COVID-19 and early treatments for secondary prevention adaptable in outpatient settings (El-Warrak et al., 2023). Rapid and easily accessible preventive strategies are needed as less than 33% world population is protected by receiving a booster dose of COVID-19 vaccines (World Health Organization, 2024a).

The human body is constituted with microbiota more than human cells, particularly in the gut. Direct interaction between gut microbiota and immune system supports each other as over 70% of body's immune cells are in the gastrointestinal tract (Nguyen et al., 2022). Growing evidence has shown the bidirectional microbial cross-talk between gut and lung, which is named the gut–lung axis (Dang and Marsland, 2019). While the respiratory tract is infected, gut microbiota activates the immune cells and migrates to the lung, modulating its immunity (Fanos et al., 2020). When severe acute respiratory syndrome coronavirus-2 (SARS-CoV-2) infects the gut, dysbiosis, which is the maladjustment of gut microbiota could occur. Dysbiosis could create uncontrolled pro-inflammatory cytokines, thus damaging the lung through cytokine storm (Gohil et al., 2021). Recent studies have shown the direct connection between microbiota and COVID-19. A cohort study done by Gou et al. (2021) revealed that dysbiosis predisposed healthy individuals to higher susceptibility and severity of COVID-19, thus restoring the gut microbiota balance could be a beneficial COVID-19 prophylaxis for healthy, especially geriatric outpatients due to their diminished diversity and metabolic activity of gut microbiome. In contrast, existing empirical antibiotics given to three-quarters of COVID-19 patients did not improve patient outcomes but prolonging systematic inflammation due to worsened dysbiosis (Xu et al., 2020).

Food and Agriculture Organization defines probiotics as live microorganisms that confer a health benefit to the host when administered in adequate amounts (Food and Agriculture Organization, 2001). Probiotics has attained much attention due to its safe prophylactic use against other respiratory coronavirus strains (Jiang et al., 2016; Morais et al., 2020). With increasing scientific evidence and demand, the global probiotics market is foreseen to grow at 14% annually (Grand view research, 2023). Probiotics was proven to confer antiviral properties via experimental and clinical studies over the past decade (Jeon et al., 2023; King et al., 2014; Zhao et al., 2022), and their use against COVID-19 has also been researched recently in various countries, including Europe (Di Pierro et al., 2022), China (Li et al., 2021), Japan (Kageyama et al., 2022), Korea (Das et al., 2021) and Russia (Sankova et al., 2022). However, existing trials in adapting probiotics for preventing intestinal and systemic effects of COVID-19 are observational, single-centre, not prospective and randomized (Ceccarelli et al., 2021; Li et al., 2021). These limit the extrapolation of outcomes to a more diverse population.

Nonetheless, studies have found that in patients infected with COVID-19, probiotics could shorten the persistence of multisystemic symptoms, so-called long-COVID (Lau et al., 2024). Incidence of long-COVID among outpatients with mild symptoms could reach up to 30% which contributes to profound health and social impacts, like functional impairments (Davis et al., 2021). For instance, in the UK, less than 1% of COVID-19 patients with functional limitations received specialist care (Prashar, 2023). Therefore, probiotics being readily accessible and easy treatment regime might help ease primary care management and alleviate public health concerns on unmet care need.

A few meta-analyses have summarized the available evidence regarding the effectiveness of using probiotics in reducing COVID-19 burden (Tian et al., 2023; Wu et al., 2023; Zhu et al., 2023). Nonetheless, most of these focus on hospitalized patients with severe COVID-19 infection with outcomes that are constrained to in-hospital settings (i.e., length of hospital stays, rate of intensive care unit admission and ventilation support). This limits our understanding on the effectiveness of probiotic use in milder COVID-19 cases who are often treated in outpatient settings (Kikkenborg Berg et al., 2022; Roessler et al., 2022). To our best understanding, this is the first narrative systematic review aiming to evaluate the effects of probiotics on preventing COVID-19 and treating COVID-19 patients with mild symptoms in outpatient settings through synthesizing existing experimental literature.

## Methods

This systematic review was conducted according to the PRISMA guidelines. We pre-registered a protocol on PROSPERO: CRD42024611230.

### Inclusion and exclusion criteria

The study selection was restricted to randomized controlled trials (RCTs) and randomized or non-randomized before-and-after interventional studies in English only. Non-randomized studies are included as subjects excluded from RCTs of preventive intervention tend to have worse prognosis and less healthy (McKee et al., 1999). Geographical limitation was not imposed. For the population of interest, participants exposed to COVID-19 or outpatients with mild COVID-19, regardless of age, gender, ethnicity, other demographics characteristics and health status were included. According to the World Health Organization, mild COVID-19 is defined as patients exhibiting signs and symptoms like cough and diarrhoea without pneumonia and can be managed at outpatient care while moderate to critical stage warrants hospitalization (World Health Organization, 2023). The intervention included all probiotics strains either alone or as an adjunct to standard care or synbiotics. Synbiotics are substrates utilized by gut microbiota to confer health benefits. Comparators could consist of placebo or standard care without supplementation of probiotics. Studies reporting efficacy of probiotics in preventing COVID-19 or reducing disease progression as outcomes were included. Primary outcomes were infection incidence and duration of symptoms. Secondary outcomes, including patients’ demographics, treatment matrix, changes in immune reaction and gut and/or upper respiratory tract (URT) microbiome were also evaluated.

Non-RCTs and animal studies were excluded. Studies involving COVID-19 patients in moderate to critical stage were excluded as our interest was in the outpatient realm. In addition, studies not including probiotics as intervention and evaluating outcomes of interest were excluded. Theses or commentary articles were also excluded.

### Search strategy

PubMed, Embase and Cochrane Library were searched from inception to May 2024. Keywords, Medical Subject Headings and text word terms for COVID-19 (e.g., SARS-CoV-2, coronavirus disease, etc.) and probiotics (e.g., synbiotics, lactobacillus) were searched. To expand database searches, lactobacillus, bifidobacterium and saccharomyces, which were the most studied probiotics strains in reducing severity of respiratory tract infection were especially searched. Also, the reference lists of all initial studies were scrutinized to further identify relevant publications that were not indexed in the databases. See Supplemental materials for details of search terms used.

### Study selection and data extraction

Two reviewers (CHHC, MMSS) independently screened all studies for inclusion and disagreements were resolved with a third reviewer (DS). All studies identified in the search were imported to EndNote library to remove duplicates. Titles and extracts of all records were first screened according to the inclusion criteria and full-text reports were retrieved and further assessed their eligibility.

The following information was extracted: (i) study design; (ii) participants characteristics; (iii) probiotics regimes and effects; (iv) outcomes; (v) compliance rate and (vi) adverse events (AEs).

### Data analysis

A narrative analysis instead of meta-analysis was conducted due to the clinical and methodological heterogeneity, like probiotics species and dosage. To enhance the veracity of the review's findings, this study adopted the Synthesis without Meta-analysis guideline in results presentation (Higgins et al., 2019). Due to the variable outcome measurements across studies, we calculated all outcomes into a standardized metrics (i.e., odds ratio (OR)) to aid interpretation. Also, structured tabulation of study results was conducted. Firstly, the studies were grouped by primary outcomes separately in two tables, that is, primary prevention and secondary prevention. Within each table, studies were re-ordered by risk of bias which aimed to emphasize on the results that influence conclusions the most.

### Risk of bias assessment

We (CHHC, MMSS) independently assessed risk of bias in randomized trials and non-randomized results by using Cochrane risk-of-bias tool (RoB 2.0) and risk of bias in non-randomized studies of interventions tool (ROBINS-I) respectively. RoB 2.0 assesses the risk of bias in five distinct domains, with classification as low, some concerns and high. ROBINS-I considers each study as if mimicking a hypothetical RCT and assesses seven domains, with classification as low, moderate, serious and critical.

### Certainty of evidence

Overall certainty of evidence for two primary outcomes was assessed using the Grading of Recommendations, Assessment, Development, and Evaluations approach. Gradings were classified as high, moderate, low or very low based on the risk of bias, inconsistency, indirectness, publication bias and imprecision. We reported overall grading of evidence for incidence of COVID-19 infection and complete remission rate in summary of findings (SoF) table adaptable in narrative review (Murad et al., 2017). Standard reporting language was used.

## Results

**Figure 1.**
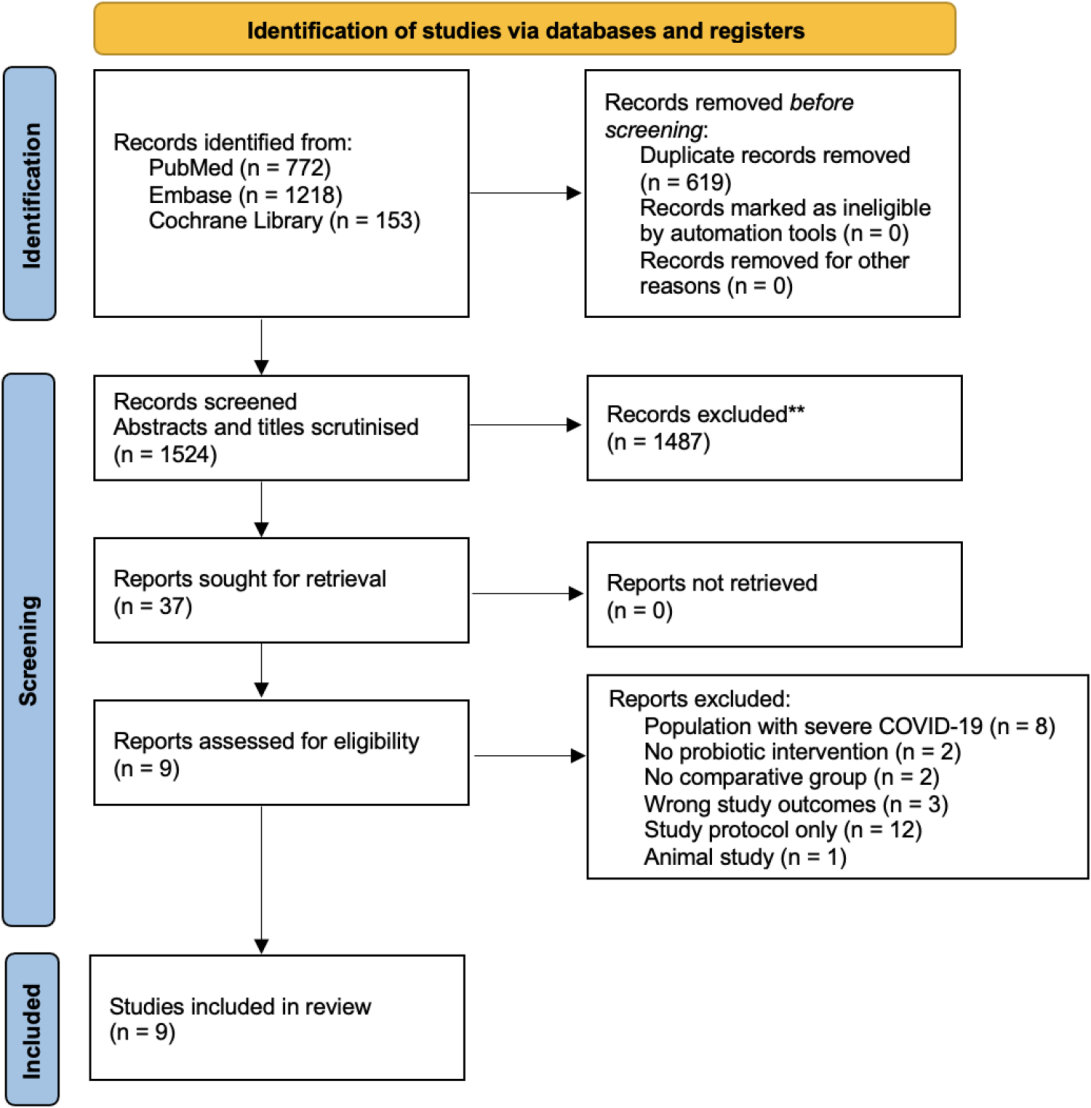
PRISMA flow diagram for study selection.

The search retrieved 4897 records of which 1125 were duplicates. After initial screening by titles and abstracts, 23 studies were analysed for inclusion criteria from which 15 studies were excluded. No article was identified by manual search from the references of included studies. A total of eight studies (Ahanchian et al., 2021; De Boeck et al., 2022; Forsgård et al., 2023; Gutiérrez-Castrellón et al., 2022; Kolesnyk et al., 2024; Mozota et al., 2021; Rodriguez-Blanque et al., 2022; Wang et al., 2021; Wischmeyer et al., 2024) were included in this systematic review (see Figure 1), consisting of eight RCTs and one pre-post study.

### Study characteristics

Two studies were from Spain (Mozota et al., 2021; Rodriguez-Blanque et al., 2022) and one each from Belgium (De Boeck et al., 2022), Sweden (Forsgård et al., 2023), Mexico (Gutiérrez-Castrellón et al., 2022), Ukraine (Kolesnyk et al., 2024), the USA (Wischmeyer et al., 2024), China (Wang et al., 2021) and Iran (Ahanchian et al., 2021). A total of 1235 participants were included, 633 in the probiotic group and 602 in the placebo group. The age of the participants ranged from below 18 to 89 year old. All participants were outpatients either exposed to SARS-CoV-2 or with mild COVID-19 confirmed by polymerase chain reaction (PCR) or physicians. Probiotics were given in variable forms of capsules (Ahanchian et al., 2021; Forsgård et al., 2023; Gutiérrez-Castrellón et al., 2022; Kolesnyk et al., 2024; Rodriguez-Blanque et al., 2022; Wischmeyer et al., 2024), throat spray (De Boeck et al., 2022), yoghurt (Mozota et al., 2021) and lozenges (Wang et al., 2021). Five RCTs used placebo capsules containing maltodextrin and vitamins as comparators, and one used standard care. Five studies examined mono-strain probiotics bacteria and three studies examined a combination of probiotics strains. Of the three genera of probiotics studied, lactobacillus species were most studied, followed by bifidobacteria and *Streptococcus*. *Lactiplantibacillus plantarum* and *Lacticaseibacillus rhamnosus* were the most common strains studied (Ahanchian et al., 2021; De Boeck et al., 2022; Gutiérrez-Castrellón et al., 2022; Kolesnyk et al., 2024; Wischmeyer et al., 2024). The prescribed doses were between 9.5 × 10^8^ and 2 × 10^10^ for 3 weeks to 4 months. See Table 1 for details on methodological characteristics of the included studies.

**Table 1.** Methodological design of included studies.

Study (country)	Study design and sample size	Characteristics of study participants	Intervention group (dosage in CFU/day)	Comparison group	Outcomes	Duration of intervention
Ahanchian et al., 2021 ^b^ (Iran)	Randomized, double-blind, placebo-controlled trial *N* = 60	Age (%): <30 (23) >30 (77) Medical staff working in COVID-19 wards	*Lactobacillus casei*, *Lacticaseibacillus rhamnosus*, *Streptococcus thermophilus*, *Bifidobacterium breve*, *Lactobacillus acidophilus*, *Bifidobacterium infantis*, *Lactobacillus bulgaricus* capsule Lactocare™ (1 × 10^9^)	Placebo capsule	Outcomes of interest: 1. Incidence of COVID-19 diagnosis 2. Duration of symptoms 3. Severity of symptoms	30 days + 45 days follow-up
De Boeck et al., 2022 ^f ^ (Belgium)	Randomized, double-blind, placebo-controlled trial *N* = 64	Age (mean ± SD): IG: 42 ± 12 years, CG: 43 ± 12 years Male/female ratio: IG: 13/21, CG: 11/19 COVID-19 outpatients with mild-to-moderate symptoms	*L. casei* AMBR2, *L. rhamnosus GG*, and *Lactiplantibacillus plantarum* WCFS1 probiotic throat spray (9.5 × 10^8^)	Placebo spray containing vitamins D_3_ and E	Outcomes of interest: 1. Severity of symptoms 2. Duration of symptoms 3. Change in airway microbiome	2 weeks + 1 week follow-up
Forsgård et al., 2023 ^g^ (Sweden)	Randomized, triple-blind, placebo-controlled trial *N* = 89	Age, mean (95% CI): IG: 51.5 (21–60) CG: 48 (29–60) Male/female ratio: IG: 7/41 CG: 8/33 COVID-19 infected participants	*Limosilactobacillus reuteri* DSM 17938 plus vitamin D3 probiotics tablets Protectis™ (2 × 10^10)^	Vitamin D3 tablets	Outcomes of interest: Anti-SARS-CoV-2 antibody response upon infection Outcomes not of interest: Vaccine-induced antibody responses	6 months
Gutiérrez-Castrellón et al., 2022 ^h^ (Mexico)	Randomized, quadruple-blind, placebo-controlled trial *N* = 300	Age, mean (95% CI): IG: 34 (26–45), CG: 39 (27–49) Male/female ratio: IG: 68/82, CG: 71/79 Symptomatic COVID-19 outpatients	*Lactiplantibacillus plantarum* KABP022, KABP023, KAPB033 and *Pediococcus acidilactici* KABP021 capsules (2 × 10^9^)	Placebo capsule containing maltodextrin	Outcomes of interest: 1. Remission rate 2. Progression to hospitalization 3. SARS-CoV-2 viral load	30 days
Kolesnyk et al., 2024 ^i^ (Ukraine)	Randomized, double-blind, placebo-controlled trial *N* = 70	Age, median (IQR): IG: 44 (36–48), CG: 46 (37–53) Male/female ratio: IG: 18/16, CG: 19/17 Symptomatic COVID-19 outpatients	*Bifidobacterium lactis*, *Bifidobacterium longum*, *L. rhamnosus*, *L. casei*, *L. acidophilus* capsule (5 × 10^9^)	Placebo capsule	Outcomes of interest: 1. Time to resolution 2. Case severity progression 3. SARS-CoV-2 IgG level	28 days
Mozota et al., 2021 ^e^ (Spain)	One group pre-post study *N* = 22	Age, mean (95% CI): 84.95 (81.41–88.49) Male/female ratio: 11/11 Elderly in nursing home	*Ligilactobacillus salivarius* MP101 in yoghurt (>10^9^)	N/A	Outcomes of interest: 1. Infection or remission of COVID-19 diagnosis 2. Nasal and faecal inflammatory profiles Outcomes not of interest: 1. Effect on functional, cognitive and nutritional status	120 days
Rodriguez-Blanque et al., 2022 ^c^ (Spain)	Randomized, double-blinded, placebo-controlled multicentre trial *N* = 255	Age (mean ± SD): IG: 41 ± 11 years, CG: 41 ± 12 years Male/female ratio: IG: 18/109, CG: 24/104 Healthcare workers caring for COVID-19 patients	*Loigolactobacillus coryniformis* K8 probiotics capsules (3 × 10^9^)	Placebo capsule containing 220 mg of maltodextrin	Outcomes of interest: 1. Incidence of COVID-19 diagnosis 2. Duration of symptoms 3. Severity of symptoms Outcomes not of interest: 1. Immune response of COVID-19 vaccine	2 months; monthly follow-up
Wischmeyer et al., 2024 ^a^ (USA)	Randomized, double-blind, placebo-controlled trial *N* = 182	Age (%): <18 (22.5) 18–64 (72) ≥65 (5.5) Male/female ratio: IG: 31/60, CG: 36/55 Exposed household contact with COVID-19 within the past 7 days	*L. rhamnosus* GG probiotics capsules (2 × 10^10)^	Placebo capsules containing 325 mg of microcrystalline cellulose	Outcomes of interest: 1. Incidence of COVID-19 diagnosis 2. Time to COVID-19 diagnosis 3. Severity of symptoms 4. Duration of symptoms 5. Change of faecal microbiome	28 days
Wang et al., 2021 ^d^ (China)	Randomized, placebo-controlled multicentre trial *N* = 193	Age (mean ± SD): IG: 36.1 ± 8.6 years, CG: 35.7 ± 8.9 years Male/female ratio: IG: 30/68 CG: 26/69 Frontline medical staff caring in close contact with COVID-19 patients	*S. thermophilus* ENT-K12 slow-dissolving oral lozenges Probionet GmbH™ (>2 × 10^9^)	No intervention	Outcomes of interest: 1. Incidence of COVID-19 diagnosis 2. Severity of symptoms Outcomes not of interest: 1. Uptake of medicine 2. Days of work absent	1 month

B.: *Bifidobacterium*, L.: *Lactobacillus*, S.: *Streptococcus*; COVID-19: coronavirus disease 2019; IgG: immunoglobulin.

Data on baseline demographics, medical history and COVID-19 infection records were collected from health-record systems and self-report. Symptomology relied on self-report and measurement tools, that is, Post-COVID Functional Scale (Kolesnyk et al., 2024).

### Risk of bias

Risk of bias assessments, conducted on nine studies, is summarized in Table 2. Three RCTs were found to be robust, scoring ‘low’ across all domains (Forsgård et al., 2023; Gutiérrez-Castrellón et al., 2022; Wischmeyer et al., 2024). All RCTs except one (Ahanchian et al., 2021) scored low in randomized process with allocation concealment. Four RCTs were assessed to have ‘some concern’ in deviation of intended interventions. Two studies had no information on participants blinding who would highly be aware of their intervention assignment (Rodriguez-Blanque et al., 2022; Wang et al., 2021). Another two studies undertook per-protocol instead of intention-to-treat analysis (De Boeck et al., 2022; Kolesnyk et al., 2024). All RCTs were at low risk in missing data with less than 5% loss to follow-up. One RCT was rated some concern in outcome measurement as participants were not blinded while reporting subjective outcomes (Wang et al., 2021). An RCT scored moderate risk in selection of the reported result as the outcomes presented in the protocol was in discordance with that in the published study (Rodriguez-Blanque et al., 2022).

**Table 2.** Assessment of risk of bias using ROB 2.0 and ROBINS-I.

Authors	Study design	1. Bias arising from randomization	2. Bias due to deviations from intended interventions	3. Bias due to missing outcome data	4. Bias in measurement of outcome	5. Bias in selection of the reported result	Overall ROB
Ahanchian et al., 2021	RCT	Some concern	Low	Low	Low	Low	Some concern
Wischmeyer et al., 2024	RCT	Low	Low	Low	Low	Low	Low
Rodriguez-Blanque et al., 2022	RCT	Low	Some concern	Low	Low	Some concern	Some concern
Wang et al., 2021	RCT	Low	Some concern	Low	Some concern	Low	Some concern
De Boeck et al., 2022	RCT	Low	Some concern	Low	Low	Low	Low
Forsgård et al., 2023	RCT	Low	Low	Low	Low	Low	Low
Gutiérrez-Castrellón et al., 2022	RCT	Low	Low	Low	Low	Low	Low
Kolesnyk et al., 2024	RCT	Low	Some concern	Low	Low	Low	Some concern

Authors	Study design	1. Bias due to confounding	2. Bias in participants selection	3. Bias in classification of interventions	4. Bias due to deviations from intended interventions	5. Bias due to missing data	6. Bias in measurement outcomes	7. Bias in results selection	Overall ROB
Mozota et al., 2021	Pre-post	Moderate	Low	Low	Low	Critical	Serious	Low	Critical

ROB 2.0: Cochrane risk-of-bias tool; ROBINS-I: risk of bias in non-randomized studies of interventions tool; RCT: randomized controlled trial.

The controlled pre-post design scored moderate in bias of confounding as residual confounding persisted without randomization (Mozota et al., 2021). Its bias of missing data was rated as critical as less than 95% of participants finished the trial (e.g., some deceased before trial) and did not address the missing data. Lastly, bias in outcome measurement was rated serious as patients were not blinded and possibly aware of the study hypothesis.

### Prevention of COVID-19 outcomes

A total of five studies (Ahanchian et al., 2021; Mozota et al., 2021; Rodriguez-Blanque et al., 2022; Wang et al., 2021; Wischmeyer et al., 2024) included incidence of COVID-19 diagnosis as their primary or secondary outcomes (see Table 3).

**Table 3.** Findings of studies with participants without COVID-19 at baseline (primary prevention).

Study (country)	Total study duration	Number of subjects’ confirmed COVID-19 (*n*/*N*, %) odds ratio (95% CI)	Reported probiotic effects	Compliance rate (*n*/*N*, %)	Adverse events (*n*/*N*, %)	Limitations/comments
Low risk of bias						
Wischmeyer et al., 2024 ^a^ (USA)	60d (28d intervention, 32d follow-up on complications upon COVID-19 diagnosis)	IG: 8/91 (8.8%) CG: 14/91 (15.4%) OR: 0.53 (0.21–1.33)	↓ Incidence of COVID-19 **↑ Prolonged time to symptoms onset ↓ COVID-19 symptoms severity** No hospitalization	110/110 (100%) ^a^110 reported back their adherence timepoint	Bloating 7/91 (7.7%) Stomach Upset 2/91 (2.2%)	Small sample size; self-reported data; potential confounding effects
Some concerns						
Ahanchian et al., 2021 ^b^ (Iran)	75d (30d intervention, 45d follow-up)	IG: 0/29 (0%) CG: 3/31 (9.68%) OR^a^: 0.14 (0.01–2.79)	↓ Incidence of COVID-19 ↓ Respiratory and gastrointestinal symptoms	60/60 (100%)	N/A	Small sample size; potential confounding effects
Rodriguez-Blanque et al., 2022 ^c^ (Spain)	2 mo (blood sampling at the end of study if subjects received COVID-19 vaccination during intervention period)	IG: 2/127 (1.6%) CG: 2/128 (1.6%) OR: 1.01 (0.14–7.27)	↓ Incidence of COVID-19 and severity of symptoms **↑ IgG level upon receiving COVID-19 vaccine after 81d ↓ Side effects of COVID-19 vaccine**	255/255 (100%)	0/255 (0%)	Wrong timing of subjects’ recruitment; low generalization (only included Caucasians)
Wang et al., 2021 ^d^ (China)	1 mo (included 1 screening visit and 1 final visit)	IG: 8/98 (8.2%) CG: 22/95 (23.2%) OR: 0.29 (0.12–0.70)	**↓ Incidence of COVID-19 ↓ Incidence and severity of key symptoms ↓ Duration of infection episodes** Optimal at least 10d regime	>90%	0/193 (0%)	Small sample size; short study period
Critical risk of bias						
Mozota et al., 2021 ^e^ (Spain)	120d	0/22	↓ Incidence and re-emergence of COVID-19 **↑ Functional and nutritional status**	≥86%	N/A	Small sample size; quasi-experimental

d: day; mo: month; OR: odds ratio.

^a^ Haldane–Anscombe correction.

**Figure 2.**
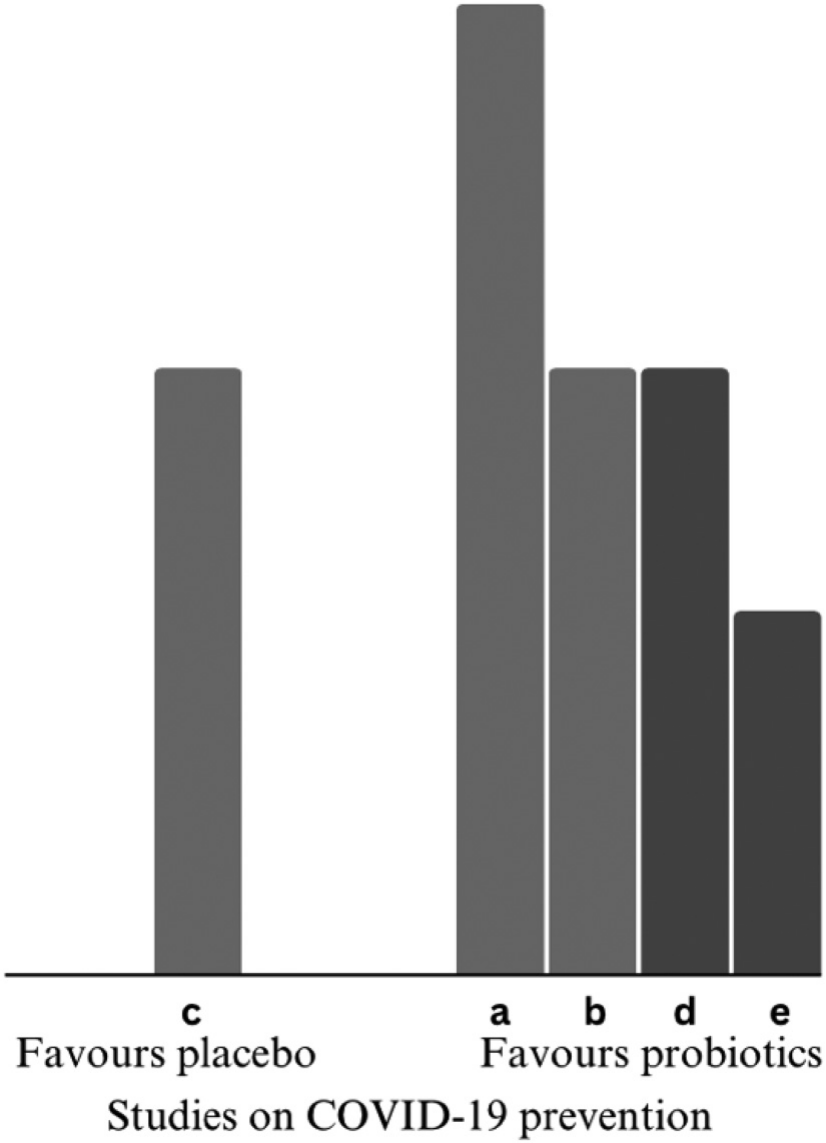
Harvest plot on primary outcome-COVID-19 incidence. Height depicts overall risk of bias (tall = low risk of bias; medium = some concerns; short = high risk of bias). Shading depicts significance of findings (light grey = insignificant; dark grey = significant). Alphabet characters represent the studies (refer to Table 3). COVID-19: coronavirus disease 2019.

Bold text denotes significant results. Alphabet characters represent respective studies shown in Figure 2.

#### Incidence of COVID-19 infection

There was evidence that probiotics had a positive effect on COVID-19 prevention, with four of five studies favouring the intervention (Ahanchian et al., 2021; Mozota et al., 2021; Wang et al., 2021; Wischmeyer et al., 2024). An RCT conducted by Wischmeyer et al. (2024) assessed the effects of 28 days probiotics as household post-exposure prophylaxis and found a reduction in COVID-19 infection, although not statistically significant. However, time to development of COVID-19 was significantly lengthened. Another RCT investigating the effects of 2-month probiotics on healthcare workers reported no effect on incidence of COVID-19 (Rodriguez-Blanque et al., 2022). In contrast, Wang et al. (2021) reported significantly large reduction in COVID-19 incidence (OR 0.29, 0.12–0.70) among healthcare workers who took oral-dissolving probiotics for 30 days. Course of infection and URT symptoms like sore throat also showed greater reduction than the control group. Similarly, results from another RCT (Ahanchian et al., 2021) suggested probiotics capsules largely reduced COVID-19 incidence and related olfactory symptoms among high-risk healthcare workers. A pre-post study (Mozota et al., 2021) conducted in the elderly home protected all participating elderly from COVID-19 re-infection by drinking probiotics-added yoghurt for 4 months. The direction of effects is presented in Figure 2.

Two studies revealed the confounding effects of age and comorbidities on incidence rate. Wischmeyer et al. (2024) found that old age significantly increased COVID-19 symptoms and infection. Following adjustment for smoking and hypertension status they found hypertension predicted more symptoms. In another study (Mozota et al., 2021) in which incidence rate reduced accompanying with cognitive and nutritional improvement was confounded by diabetes status of elderly.

#### Change in microbiome and immunological markers

Wischmeyer et al. (2024) found that individuals who received probiotics showed greater abundance of same bacterial strain in the stool sample than those in the placebo group. While Mozota et al. (2021) studied immunological markers in nasal and stool samples, significant change of interleukins was observed. As some participants received the COVID-19 vaccines amid the intervention period, Rodriguez-Blanque et al. (2022) measured the antibody response and reported a significant higher immunoglobulin G (IgG) level in probiotics group after 81 days from the first dose. Similarly, Wang et al. (2021) estimated a sustained protective effect after 10 days of probiotics intake due to oral colonization of probiotics strains.

### Treatment of mild COVID-19 outcomes

A total of four studies (De Boeck et al., 2022; Forsgård et al., 2023; Gutiérrez-Castrellón et al., 2022; Kolesnyk et al., 2024) included complete symptomatic remission or viral clearance as their outcomes (see Table 4).

**Table 4.** Findings of studies with participants with mild COVID-19 at baseline (secondary prevention).

Study (country)	Total study duration	COVID-19 positivity rate after study period (*n*/*N*, %) odds ratio (95% CI)	Reported probiotic effects	Compliance rate (*n*/*N*, %)	Adverse events (*n*/*N*, %)	Limitations/comments
Low risk of bias						
De Boeck et al., 2022 ^f^ (Belgium)	3w (weekly nose-throat swab sampling)	IG: 2/30 (6.7%) CG: 7/27 (26%) OR: 0.20 (0.04–1.09)	↓ COVID-19 positivity rate ↑ Anti-SARS-CoV-2 IgG **↑ Upper airway microbiome ↓ Duration of acute symptoms**	95%	Unpleasant taste of spray	Small sample size; delay of treatment
Forsgård et al., 2023 ^g^ (Sweden)	6mo (blood-taking at 1, 3, 6 mo)	IG: 6/48 (12.5%) CG: 6/41 (14.6%) OR: 0.83 (0.25–2.82)	↑ Yet non-significant anti-S IgG/IgA, anti-RBD IgG/IgA (potentially ↓ Breakthrough infection), and virus neutralizing antibody level **↑ Serum IgG upon vaccination in longer term (**≥**28d)**	90%	Gastrointestinal complaints 3/89 (3.4%)	Small sample size; irregular sampling visits/trial started prior to infection
Gutiérrez-Castrellón et al., 2022 ^h ^ (Mexico)	30d (site visit at day 0, 15 and 30)	IG: 69/147 (46.9%) CG: 105/146 (71.9%) OR: 0.35 (0.21–0.56)	**↑ Complete remission ↓ Duration of symptoms, e.g., fever, loose stool ↓ Viral load and inflammatory markers**	283/300 (94.3%)	IG: 41/150 (27.3%) CG: 63/150 (42.0%) cough, body aches, etc.	Single-centre; generalize to population below 60
Some concerns						
Kolesnyk et al., 2024 ^i^ (Ukraine)	28d (3 follow-ups)	Time to resolution: IG: 11d, CG: 14d *p* = 0.035	**↓ Constitutional and respiratory symptoms ↓ Scores on severity scale** ↑ **RBD/S antibody production ↓ Medicine intake, i.e., throat antiseptics**	>89%	Rash 2/34 (5.9%)	Potential confounders; invalidated questionnaires

RBD: receptor binding domain; S: spike; IgG: immunoglobulin G; IgA: immunoglobulin A; d: day, mo: month, w: week; OR: odds ratio.

**Figure 3.**
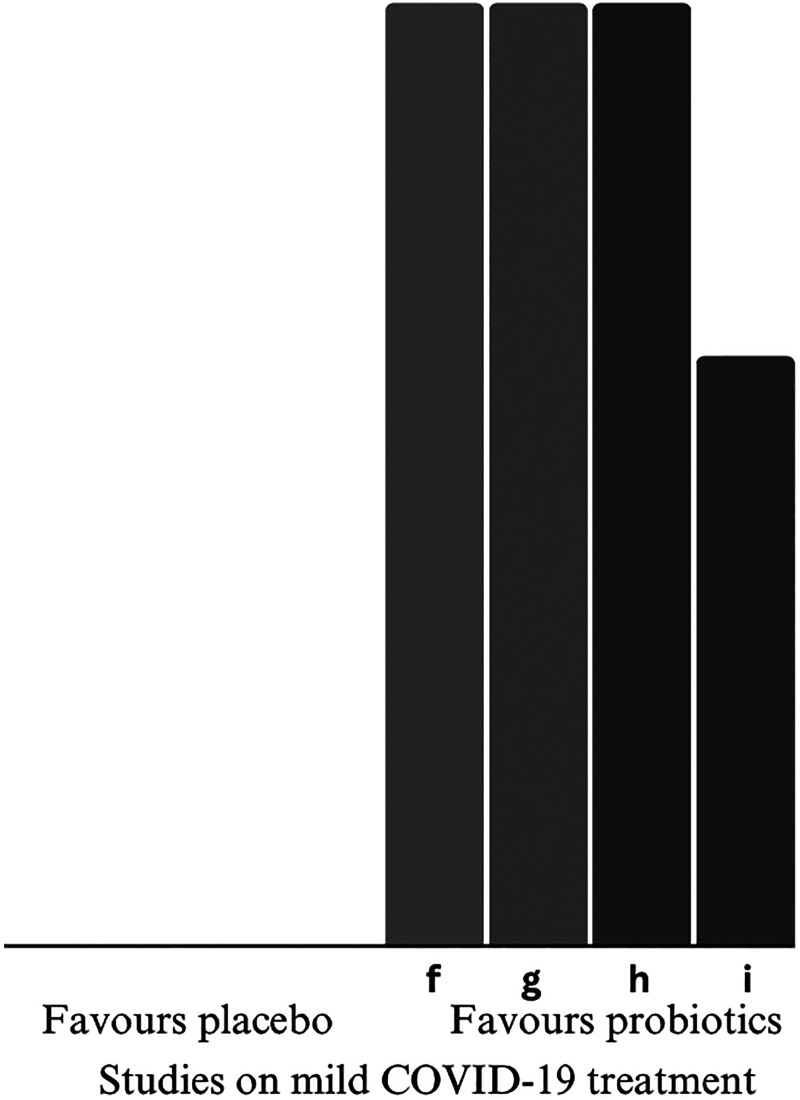
Harvest plot on primary outcome-mild COVID-19 remission. Height depicts overall risk of bias (tall = low risk of bias; medium = some concerns; short = high risk of bias). Shading depicts significance of findings (light grey = insignificant; dark grey = significant). Alphabet characters represent the studies (refer to Table 4). COVID-19: coronavirus disease 2019.

Bold text denotes significant results. Alphabet characters represent respective studies shown in Figure 3.

#### Complete remission rate

All studies favoured the intervention, manifesting statistical trend in increasing mild COVID-19 complete recovery in the probiotic group than the placebo group. Duration of ambulatory symptoms was shortened in outpatients who took probiotics. The common reported symptoms included systematic (i.e., fatigue, fever) (De Boeck et al., 2022; Forsgård et al., 2023; Gutiérrez-Castrellón et al., 2022; Kolesnyk et al., 2024), respiratory (i.e., cough) (De Boeck et al., 2022), gastrointestinal (i.e., diarrhoea) (Gutiérrez-Castrellón et al., 2022) and neurological (i.e., anxiety). The direction of effects is presented in Figure 3. Gutiérrez-Castrellón et al. (2022) found the significant remission rate was confounded by metabolic comorbidities, including diabetes and obesity.

#### Change in microbiome and immunological markers

De Boeck et al. (2022) indicated that rich nasal abundance of lactobacilli significantly reduced acute symptoms by using probiotics-containing throat spray. In contrast, Gutiérrez-Castrellón et al. (2022) found no change in gut microbiome despite significant reduction of COVID-19 positivity in the probiotic group. They instead showed enterotypes, the initial gut microbial composition contributed to the significant result. Also, serum IgG and immunoglobulin M peaked at day 30 and inflammatory markers plummeted at day 15. Besides, two studies found a statistical increase of anti-spike IgG and anti-receptor binding domain (Forsgård et al., 2023; Kolesnyk et al., 2024).

### Adverse events

The most common AEs in the probiotic groups were gastrointestinal-related, including bloating and abdominal pain. Non-gastrointestinal AEs included cough, body ache and unpleasant taste. One study (Gutiérrez-Castrellón et al., 2022) found the highest rate of AEs at 27.3% in the probiotics group. After comparing the probiotics and placebo groups, authors found the AEs was 35% lower with probiotic as compared to the placebo group and were mostly due to natural symptom flares. Besides, C-reactive protein (CRP) continuously declined along the intervention which could predict lower risk of multiorgan impairment in outpatient care (Dennis et al., 2021). Although probiotics has been used in septic patients (Shimizu et al., 2018), this review has small sample size on high-risk populations and AEs was not reported in the study targeting the elderly (Mozota et al., 2021); therefore, the safety profile could not be generalized.

### Certainty of evidence

The quality of evidence (QoE) was ‘moderate’ for reducing incidence of COVID-19. QoE was downgraded by one for the risk of methodological bias in which only one out of five studies scored low in risk of bias and its sample size was relatively small. The remaining trials lacked blinding which further led to measurement bias in subjective outcomes. The QoE was further downgraded by one for inconsistency as the magnitude of effects varied across studies with wide confidence intervals. Lastly, QoE was upgraded by one due to the presence of dose–response gradient between probiotics and humoral immune responses.

The QoE was ‘moderate’ for remission of mild COVID-19. Although all studies favoured the intervention, magnitude of effects differed where two out of four RCTs showed significant effects. The remaining studies presented considerably wide effect estimates. SoF is presented in Table 5.

**Table 5.** Summary of findings table for primary outcome by GRADE.

Probiotics compared to placebo for outpatient COVID-19			
**Patients or population:** mild COVID-19			
**Settings:** outpatient care			
**Intervention:** probiotics capsules, throat spray or drinks			
**Comparison:** placebo capsules			

Outcomes	Impact	Number of participants (studies)	Certainty of the evidence (GRADE)*
Incidence of COVID-19	Most studies showed small reduction in COVID-19 infection upon exposure.	712 (4 RCTs, 1 pre-post study)	_⊕⊕⊕⊖abc_ Moderate^†^
Complete remission without severe progression	Most studies showed big difference where probiotics quickened complete COVID-19 recovery and improved severity of symptoms.	523 (4 RCTs)	_⊕⊕⊕⊖b_ Moderate^†^

*GRADE: grade working group grades of evidence:

**High** = This research provides a very good indication of the likely effect. The likelihood that the effect will be substantially different^†^ is low.

**Moderate** = This research provides a good indication of the likely effect. The likelihood that the effect will be substantially different^†^ is moderate.

**Low** = This research provides some indication of the likely effect. However, the likelihood that it will be substantially different^†^ is high.

**Very low** = This research does not provide a reliable indication of the likely effect. The likelihood that the effect will be substantially different^†^ is very high.

COVID-19: coronavirus disease 2019; RCT: randomized controlled trials.

^a^ Serious risk of bias due to inadequate blinding and bias in outcome measurement

^b^ Serious inconsistency due to heterogeneity of results (likely due to wide CIs).

^c^ Presence of dose–response gradient.

## Discussion

### Summary

This systematic review aimed to comprehensively evaluate the effectiveness of probiotics in the prevention and treatment of mild cases of COVID-19 in outpatient settings. We found relatively few studies addressing this specific research question, and the overall quality of the available evidence was only moderate. All studies were rated low to some concern of bias except one pre-post study being critical. It is still included in this review as it is the only study investigating the vulnerable age group, that is, elderly. With its evidence of promise in accord with previous studies, this may be a starting point for implementing stronger interventions with more robust evaluations in future. Overall, the results indicate that the application of probiotics reduces the incidence of COVID-19 among individuals in various age groups and with comorbidities, showing sustained protective effects. Probiotics also accelerate clearance of symptoms in a way to enhance complete recovery from mild COVID-19 without disease progression. Although there are discrepancies in the results on the changes of gut microbiome composition, all the studies included in this review suggest the role of probiotics as easily accessible immunoregulator to be used in the outpatient care of COVID-19.

### Comparison to other literature

Our results support that probiotics prevents the progression of COVID-19 more effectively in outpatients than inpatient care. A previous study showed that hospitalized patients treated with probiotics had even prolonged inpatient stay as they were treated with antiviral drugs simultaneously which reduced the effectiveness of probiotics (Li et al., 2021). In contrast, this review showed that probiotics reduced the infection course of COVID-19 significantly, where nobody in the probiotic group took antiviral drugs nor antibiotics concurrently as they were relatively healthier with reduced need in combinational drug therapy commonly seen in hospitalized patients (Wang et al., 2021). This review is consistent with previous inpatient studies, probiotics benefited the most in elderly. Reduction of pro-inflammatory cytokines and enhancement of anti-inflammatory cytokines were significant among the community-dwelling elderly who had greater degree of dysbiosis due to ageing after consuming probiotics (Mozota et al., 2021). Similarly, an inpatient study showed that the aged taking the probiotics had strengthened anti-inflammatory response, that is, CRP reduction and thus preventing COVID-19 progression to severe stage (Ceccarelli et al., 2021). Yet, the treatment effect was higher in outpatient than inpatient settings (Ceccarelli et al., 2021; Mozota et al., 2021; Wischmeyer et al., 2024).

Outpatients receiving *Lacticaseibacillus rhamnosus* probiotics may benefit from prolonging time to symptoms onset and suffered less change in taste. A previous study found that the prolonged asymptomatic period upon exposure to SARS-CoV-2 could lead to hyperinflammatory state and severe stage of disease (Endam et al., 2020). However, in this review, no severe COVID-19 case was found probably due to probiotics in enhancing respiratory epithelium functions inside the nose, reducing replication of SARS-CoV-2 and thus its damage on olfactory cells, that is, anosmia (Ahanchian et al., 2021; Endam et al., 2020; Wischmeyer et al., 2024). The colonization of probiotics bacteria not only prevents the infection of COVID-19 by decreasing the oropharyngeal viral load, but also reducing the transmission of COVID-19 (De Boeck et al., 2022). Despite the small sample size, this review found a possible tripartite crosstalk between probiotics, circadian rhythms and COVID-19 infection as supported with recent studies (Ahanchian et al., 2021; Sengupta et al., 2021).

Furthermore, this review suggested that the baseline comorbidities of participants had confounded the positive yet insignificant protective effects of probiotics. Participants who reported more symptoms in the placebo group had three-fold more hypertension status than the probiotics group (Wischmeyer et al., 2024). In line with previous studies, hypertensive individuals who were treated with angiotensin-converting enzyme inhibitor in lowering their blood pressure had greater gut dysbiosis and weakened immunity, thus suffering from more symptoms (Yang et al., 2020). Administration of probiotics on hypertensive individuals could have generated stronger beneficial effects in strengthening host immunity and clearing of SARS-CoV-2 by dual properties of gut microbiome restoration and lowering of blood pressure as probiotics release bioactive peptides in dilating human vessels (Rajput et al., 2021). Together with hypertension, diabetes also confounded probiotics in preventing COVID-19, especially among the elderly in this review findings who showed significant improvement in nutritional intake and mobility after consuming probiotics. This ties well with previous studies wherein diabetic individuals had distinct gut microbiome from healthy ones. Lower amount of *Bacteroidetes* lineage and more of *Firmicutes* in the gut predisposed diabetic patients to malnutrition and weakened immunity, especially due to malabsorption of protein after contacting COVID-19 (Ley et al., 2006).

This review manifested the immunomodulatory mechanism of probiotics. Probiotics modulate innate immunity, distinguished by early recruitment of macrophages, dendritic cells and Natural Killer cells which are essential for viral elimination (Infusino et al., 2020; Kurian et al., 2021). This review, in accord with previous studies, supported a potent Interferon-α immune response towards respiratory viruses (Mozota et al., 2021; Sugimura et al., 2015). Probiotics could further stimulate humoral immunity which produces immunoglobulins in eliminating the virus (Akatsu et al., 2013; Kolesnyk et al., 2024; Rodriguez-Blanque et al., 2022). Low-grade chronic inflammation is observed in individuals with metabolic disorders like diabetes. Butyrate, a probiotics’ metabolite, reduces pro-inflammatory mediators to prevent overreactive immune reaction which diminishes lung injuries and pneumonia among COVID-19 patients (Kurian et al., 2021), including CRP (Gutiérrez-Castrellón et al., 2022) and tumour necrosis factor (Mozota et al., 2021). Reduction of oxidative stress also helps lowering insulin resistance and increasing appetite as well among the elderly (Mozota et al., 2021; Tao et al., 2020).

In view of the extensive community study called the PRINCIPLE done in the UK, it showed that antibiotics could not reduce risk of infection on individuals with comorbidities but worsening their gut dysbiosis (Butler et al., 2021). Hence, probiotics are effective in preventing COVID-19 and might act as extra immune booster on outpatients with comorbidities. However, to inform the best public health decision, absolute risk difference of high-risk groups with various comorbidities should be considered on optimal regime, timing and need prioritization for probiotics intake as COVID-19 prevention (Park et al., 2024).

As for using probiotics as secondary prevention of mild COVID-19, it is noted that some studies failed to show significant differences between the probiotics and placebo groups, that is, between-group effects (De Boeck et al., 2022; Forsgård et al., 2023). It can be speculated that might be due to choice of placebo, where the included studies selected vitamin D as placebo that may up-regulate the immunity and diluted the beneficial effects attributed to probiotics. The anti-inflammatory effects of vitamin D were proved to alleviate COVID-19 severity (Daneshkhah et al., 2020). It is noteworthy that synergic effect with an enhancement of 25% was found between probiotics and vitamin D (Jones et al., 2013). Hence, the result should be taken with caution due to confounders like vitamin D and levels of sun exposure (Lee et al., 2018). Furthermore, half of the included studies which were shown useful in secondary prevention of mild COVID-19 used the probiotic strain *L. plantarum*. *L. plantarum* is a bacterium originated from plants and especially rich in fermented plant food like pickles (Kageyama et al., 2022). Plant proteins act synergically with probiotics in secreting short-chain fatty chains for vital immunomodulatory functions (Seethaler et al., 2022). In this review, participants in the probiotic group who took fibre-rich diet showed stronger immune response and quicker recovery from mild COVID-19. In fact previously, a population study reported that plant-based eaters were less likely to be infected with COVID-19 than meat eaters possibly due to beneficial plant substance (Acosta-Navarro et al., 2024). Interestingly, this review is the first study in observing the potential roles of micronutrient and plant-based diets in synergizing the treatment effects of probiotics on COVID-19. The association is worth further investigation as probiotics and plant-based diets could be easily adopted by outpatients with mild COVID-related symptoms especially when fermented food products are widely available in the market, for instance, lactic acid drink could be accessed at low price in Japan as it being one of the largest producers globally (Chen et al., 2023).

In contrary to previous studies that probiotics increased diversity of gut microbiota (Biliński et al., 2022), this review reported no change in the gut but inside the nasal cavity (Gutiérrez-Castrellón et al., 2022). It could be explained that immune cells stimulated by probiotics in the gut travelled to the nose, eliciting strong immunoglobulin A antibodies response due to rich mucosal tissue located there (Sheikh Mohamed et al., 2022). Hence, nasal intake of probiotics could directly inject the microbial strains into the respiratory tract for stronger immune response (Enaud et al., 2020).

### Strengths and limitations of the review

This systematic review possesses certain strengths. Firstly, the methodological quality of the RCTs included in the review increases the reliability of results. Besides, majority of COVID-19 cases were confirmed by PCR, eliminating the risk of under/over reporting and thus strengthening the internal validity.

As for the limitations, the small sample size might have led to the lack of significant results as all included studies could not attain the pre-study sample size estimates. Secondly, the diversified settings make analysis of effects of specific probiotics strains impossible. Some studies used probiotics mixture and with other elements, that is, vitamin D. No subgroup analysis was possible to assess specific strain on relatively high-risk outpatient group as most studies generally included participants by age capped at 60 and job natures. Hence, the substantial heterogeneity contributed to underdetermination of optimal doses, strains and course of probiotics on prevention of COVID-19. Generalization of findings to wider population is difficult as between-study differences could not be extrapolated to within-study differences. Regarding the methodological limitation of the literature search, language bias exists as non-English publications might be missed. Three studies received industry funding but they all rated low-risk of bias.

Nevertheless, this review includes many robust studies which serve as a direction. Future studies with larger scales are needed to standardize probiotics strains, dosages and duration, preferably with stratification of higher risk populations, like with different comorbidities so as to assess their AEs as well. Synergic effects of different strains and supplementation, for example vitamins and booster vaccines could be investigated.

## Conclusion

This review provides evidence that probiotics are effective to prevent COVID-19 and induce remission in outpatients with mild disease. It also indicates that individuals with comorbidities and/or on plant-based diet could gain extra benefits from probiotics supplements. The practical implication of this review is that probiotics as primary and secondary intervention of COVID-19 could be given under primary care providers guidance in outpatient settings. Although this review provides promising findings on probiotics effectiveness in community settings, future research is warranted to explore optimal therapeutic regimes applicable in diverse populations.

## Supplemental Material

sj-pdf-1-nah-10.1177_02601060251378200 - Supplemental material for Effectiveness of probiotics on COVID-19 prevention and treatment against mild COVID-19 in outpatient care: A systematic review Supplemental material, sj-pdf-1-nah-10.1177_02601060251378200 for Effectiveness of probiotics on COVID-19 prevention and treatment against mild COVID-19 in outpatient care: A systematic review by Chung Hang Hannah Chau, Denes Stefler and Michelle Man Sum Szeto in Nutrition and Health

## Abbreviations

AEs: adverse events
B.: *Bifidobacterium*
COVID-19: coronavirus disease-2019
CRP: C-reactive protein
GRADE: Grading of Recommendations, Assessment, Development and Evaluations
L.: *Lactobacillus*
PCR: polymerase chain reaction
QoE: quality of evidence
RCTs: randomized controlled trials
RoB: Cochrane risk-of-bias tool
ROBINS-I: risk of bias in non-randomized studies of interventions tool
SARS-CoV-2: severe acute respiratory syndrome coronavirus-2
SoF: summary of findings
S.: *Streptococcus*
URT: upper respiratory tract
