# Supplementary material for: Effectiveness of probiotics on COVID-19 prevention and treatment against mild COVID-19 in outpatient care: A systematic review
Source: Nutr Health. 2025 Sep 26;32(2):465–80. doi: 10.1177/02601060251378200 (PMC13096631; doi:10.1177/02601060251378200)
Supplement: sj-pdf-1-nah-10.1177_02601060251378200 - Supplemental material for Effectiveness of probiotics on COVID-19 prevention and treatment against mild COVID-19 in outpatient care: A systematic review [file sj-pdf-1-nah-10.1177_02601060251378200.pdf]

# *Effectiveness of Probiotics on COVID-19 Prevention and Treatment Against Mild COVID-19 in Outpatient Care: A Systematic Review*

European Journal of Nutrition

Author[1]: Chung Hang Hannah Chau

Affiliation: Institute of Epidemiology and Health Care, University College London, UK

Email: chung.chau.23@ucl.ac.uk

Author[2]: Denes Stefler

Affiliation: Institute of Epidemiology and Health Care, University College London, UK

Email: denes.stefler@ucl.ac.uk

Author[3]: Michelle Man Sum Szeto

Affiliations: Nuffield Department of Population Health, University of Oxford, United Kingdom

Email: man.sze-to@dph.ox.ac.uk

Corresponding author: Chung Hang Hannah Chau

Correspondence email: chung.chau.23@ucl.ac.uk

## *Supplementary Information*

### PubMed Search Term and Strategy

**((COVID-19[MeSH Terms]) OR (SARS-CoV-2[MeSH Terms]) OR (Coronavirus[MeSH Terms]) OR (Severe Acute Respiratory Coronavirus 2[Title/Abstract]) OR (coronavirus disease 2019[Title/Abstract]) OR (novel coronavirus[Title/Abstract]) OR (COVID-19[Title/Abstract]) OR (COVID19[Title/Abstract]) OR (SARS-CoV-2[Title/Abstract]) OR (SARS-CoV2[Title/Abstract]) OR (Coronavirus[Title/Abstract])) AND ((Probiotics[MeSH Terms]) OR (Synbiotics[MeSH Terms]) OR (probiotic*[Title/Abstract]) OR (lactobacill*[Title/Abstract]) OR (bifidobacteri*[Title/Abstract]) OR (synbiotic*[Title/Abstract]) OR (saccharomyce*[Title/Abstract]) OR (live microbia*[Title/Abstract]))**

### Embase Search Term and Strategy

| | **Descriptors** |
|---|---|
| **#1** | exp coronavirus disease 2019/ OR exp Severe acute respiratory syndrome coronavirus 2/ OR 'COVID-19'.ab,kf,ti. OR 'COVID19'.ab,kf,ti. OR 'SARS-CoV-2'.ab,kf,ti. OR 'SARS-CoV2'.ab,kf,ti. OR 'coronavirus disease 2019'.ab,kf,ti. OR 'novel coronavirus'.ab,kf,ti. OR 'Severe Acute Respiratory Coronavirus 2'.ab,kf,ti. |
| **#2** | exp probiotic agent/ OR exp synbiotic agent/ OR 'probiotic*'.ab,kf,ti. OR 'synbiotic*'.ab,kf,ti. OR 'lactobacill*'.ab,kf,ti. OR 'bifidobacteri*'.ab,kf,ti. OR 'saccharomyce*'.ab,kf,ti. OR 'live microbia*'.ab,kf,ti. OR 'probiotic agent'.ab,kf,ti. |
| **#3** | **#1** AND **#2** |

### Cochrane Library Search Term and Strategy

| | **Descriptors** |
|---|---|
| **#1** | MeSH ("COVID-19") OR MeSH ("SARS-CoV2") OR ("Severe Acute Respiratory Coronavirus 2"):ti,ab,kw OR ("coronavirus disease 2019"):ti,ab,kw OR ("novel coronavirus"):ti,ab,kw OR ("COVID-19"):ti,ab,kw OR ("COVID19"):ti,ab,kw OR ("SARS-CoV-2"):ti,ab,kw OR ("SARS-CoV2"):ti,ab,kw |
| **#2** | MeSH ("probiotics") OR MeSH ("synbiotics") OR ("probiotic*"):ti,ab,kw OR ("synbiotic*"):ti,ab,kw OR ("lactobacill*"):ti,ab,kw OR ("bifidobacteri*"):ti,ab,kw OR ("saccharomyce*"):ti,ab,kw |
| **#3** | **#1** AND **#2** |
